# I-AUV Docking and Panel Intervention at Sea

**DOI:** 10.3390/s16101673

**Published:** 2016-10-12

**Authors:** Narcís Palomeras, Antonio Peñalver, Miquel Massot-Campos, Pep Lluís Negre, José Javier Fernández, Pere Ridao, Pedro J. Sanz, Gabriel Oliver-Codina

**Affiliations:** 1 Centre d’Investigació en Robótica Submarina (CIRS), Computer Vision and Robotics Institute, Universitat de Girona, Girona 17071, Spain; pere@eia.udg.edu; 2 Interactive and Robotic Systems Laboratory (IRSLab), Universitat Jaume I, Castelló 12071, Spain; penalvea@uji.es (A.P.); fernandj@uji.es (J.J.F.); sanzp@uji.es (P.J.S.); 3 Systems Robotics and Vision Group, Universitat de les Illes Balears, Palma de Mallorca 07122, Spain; miquel.massot@uib.cat (M.M.-C.); pl.negre@uib.cat (P.L.N.); goliver@uib.cat (G.O.-C.)

**Keywords:** autonomous underwater vehicles, manipulation, underwater intervention, field robotics

## Abstract

The use of commercially available autonomous underwater vehicles (AUVs) has increased during the last fifteen years. While they are mainly used for routine survey missions, there is a set of applications that nowadays can be only addressed by manned submersibles or work-class remotely operated vehicles (ROVs) equipped with teleoperated arms: the intervention applications. To allow these heavy vehicles controlled by human operators to perform intervention tasks, underwater structures like observatory facilities, subsea panels or oil-well Christmas trees have been adapted, making them more robust and easier to operate. The TRITON Spanish founded project proposes the use of a light-weight intervention AUV (I-AUV) to carry out intervention applications simplifying the adaptation of these underwater structures and drastically reducing the operational cost. To prove this concept, the Girona 500 I-AUV is used to autonomously dock into an adapted subsea panel and once docked perform an intervention composed of turning a valve and plugging in/unplugging a connector. The techniques used for the autonomous docking and manipulation as well as the design of an adapted subsea panel with a funnel-based docking system are presented in this article together with the results achieved in a water tank and at sea.

## 1. Introduction

Maintenance of permanent observatories, submerged oil wells, cabled sensor networks, pipes, deployment and recovery of benthic stations, or search and recovery of black boxes are examples of tasks that, nowadays, require the use of work-class remotely operated vehicles (ROVs) [1,2] deployed from dynamic positioning (DP) vessels. The cost of operating these vehicles is huge and, moreover, it requires the adaptation of these facilities to allow its manipulation from heavy ROVs. In contrast, autonomous underwater vehicles (AUVs) [3,4] are much less costly to operate, but, nowadays, their capabilities are almost restricted to survey applications. To face these intervention applications, researchers have tried to increase the autonomy of underwater systems. Pioneering works appeared in the 1990s with OTTER [5], ODIN [6], UNION [7], and AMADEUS [8]. However, it was not until the first decade of the 21st century that field demonstrations arrived.

Successful approaches were based on hybrid ROV/AUV concepts like the one proposed by the SWIMMER project [9], where an AUV shuttle transporting an ROV autonomously homes and docks into a seabed docking station. Next, the ROV, which is connected through the docking device to a remote operation station, is teleoperated during the intervention. The system avoids the need for a DP capable ship with the consequent savings. Another hybrid concept that has appeared lately is the hybrid ROV (HROV) [10,11]. These vehicles are essentially AUVs reconfigurable as ROVs when tethered through an optical fiber umbilical. Thanks to its ultra light umbilical, HROVs may also be operated from ships of opportunity without DP. When plugged, HROVs behave as conventional ROVs avoiding some of the difficulties related to the cable. Moreover, they have the capability of detaching the cable and surfacing autonomously. The most advanced demonstration to date showed a wireless teleoperated intervention using a multimodal opto/acoustic communication system from the HROV to an underwater gateway connected to an umbilical [11].

Nevertheless, all these systems keep the human within the control loop. The first fully autonomous intervention at sea was demonstrated by the ALIVE project [12], where a hovering capable AUV was able to home to a subsea intervention panel using an imaging sonar, and then, dock into it with hydraulic grasps using visual feedback. Once attached to the panel, a very simple manipulation strategy (fixed-base manipulation) was used to open/close a valve. First, object manipulation from a floating vehicle (an I-AUV) was achieved in 2009 within the Semi-Autonomous Underwater Vehicle for Intervention Missions (SAUVIM) project [13]. The capability of searching for an object whose position was roughly known a priori was demonstrated. The robot was autonomously located and the object was endowed with artificial landmarks. The vehicle hooked the object, while hovering, with a recovery device. Finally, the first underwater multipurpose object search and recovery strategy was demonstrated in the TRIDENT FP7 project in 2012. First, the object was searched using a down-looking camera and photo-mosaicking techniques. Next, how to autonomously hook the object in a water tank [14] was demonstrated. The experiment was repeated in a harbor environment using a 4 degrees of freedom (DoF) arm [15], and later with a 7 DoF arm endowed with a three-fingered hand [16]. To reduce costs and time of field experiments, some authors have opted to use simulation and control systems prototyping. In [17], the authors use a combination of software tools to model, simulate and test the control algorithms.

Given the importance of inspection maintenance and repair (IMR) tasks for the offshore industry, representative tasks usually performed by ROVs, like “Valve Turning” and “Connector Plug in/Unplug”, have been automated with different approaches. Fully autonomous fixed-base manipulation of a valve has been demonstrated in [12]. In this case, a mechanical scanning imaging sonar was used to locate and home to a subsea panel using visual servoing techniques for docking the vehicle with two hydraulic grasps. Once the vehicle was rigidly attached to the docking structure, a hydraulic 7 DOF manipulator was used to open/close a valve. The work done by the authors in the PANDORA project demonstrated autonomous free-floating valve-turning operations on a subsea panel using a Learning by Demonstration paradigm [18]. This work was extended later on to demonstrate persistent free-floating manipulation [19]. A light weight I-AUV was setting different configurations in a valve panel, according to a predefined plan, for more than 3 h performing more than 30 manipulation operations without human intervention. Although the experiment was carried out in a water tank, artificial water currents were generated and additional disturbances were introduced to test the system persistence.

The TRITON project has focused its efforts on demonstrating intervention capabilities in a submerged intervention panel connected to a permanent underwater observatory. A mock-up panel, first placed in a water tank and later at sea, has been used in different demonstrations that include: Docking to an adapted subsea panel and performing a fixed-based manipulation consisting of valve turning and connector plugging in/unplugging. Thus, this article details how the panel has been adapted to simplify the intervention, which are the alternatives to approaching the panel and how the autonomous docking/undocking maneuver and the fixed-based manipulation have been tackled.

After this introduction, the funnel-based docking station designed for this project is presented. The localization system used to navigate towards the panel and to know the relative position between the panel and the vehicle is discussed in Section 3. The strategy adopted for the docking maneuver is detailed in Section 4. Section 5 and Section 6 explain how to detect the panel elements (i.e., valve and connector) and how to initialize the arm and recalibrate on-line the end-effector position. Finally, the valve turning and the connector plugging in/unplugging procedures are described in Section 7. The paper finalizes reporting the results, first in a water tank and later at sea, obtained with Girona 500 I-AUV (University of Girona, Girona, Spain) in Section 8 just before the conclusions.

## 2. I-AUV Friendly Docking and Manipulation Station

Two approaches can be followed to perform a panel intervention with an underwater vehicle: a free-floating manipulation or a fixed-base manipulation. Although the latter requires an additional step to dock, once the vehicle is docked, the manipulation becomes easier, especially in the presence of underwater currents. Then, to allow a fixed-base manipulation, it is necessary to design an I-AUV friendly panel that simplifies the docking step. A popular solution adopted by most of ROV-friendly panels is to include several handles in which the ROV can be attached using an additional manipulator. However, to avoid this extra actuator and keep the docking maneuver as simple as possible, we have designed a friendly intervention panel based on the deliverables of the FREESUBNET network [20]. The solution adopted for these deliverables was the installation of funnel-shaped receptacles in the panel and a matching set of probes in the intervention vehicle. Funnel devices are attached to the top part of the docking structure and distributed to match the three probes mounted on the frame of the Girona 500 I-AUV [21]. When the probes are inside the funnel-shaped receptacles, the vehicle remains docked while it is exerting a forward thrust and undocks just reverting this thrust. An a priori known texture-rich flat panel is placed between the funnels to estimate the relative distance between the vehicle and the intervention panel using a vision-based system. Because vision-based localization works only on the panel vicinities, an acoustic transponder is attached to it to allow a longer range detection. Two methods has been used to acoustically detect the panel: using an ultra-short base line (USBL) system to obtain the transponder absolute position and implementing a sum of Gaussian to obtain the transponder position using only range measurements [22]. The first solution is the one implemented in this article.

**Figure 1:**
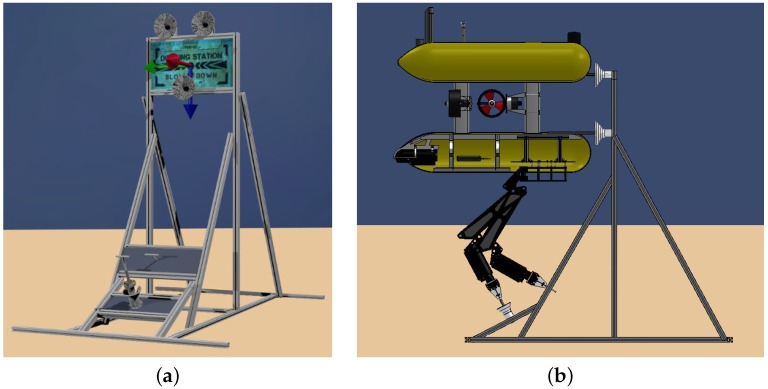
(**a**) schema of the mock-up intervention panel indicating its axis: *X* in **red**, *Y* in **green** and *Z* in **blue**; (**b**) Girona 500 I-AUV docked in the mock-up panel.

Two additional panels are placed on the lower part of the structure. These contain the mock-ups of a 1/4 turn valve and a funnel shaped hot stab connector used to demonstrate the intervention capabilities. Figure 1 shows the designed intervention panel with and without the Girona 500 I-AUV docked to it.

## 3. Vehicle and Panel Localization

Despite both the panel and the AUV including an acoustic beacon for localization purposes, it is necessary to design a system able to improve this information in order to obtain the necessary accuracy to perform a docking. The main goals of the proposed system are: (i) integrating updates from an absolute positioning system like the ones provided by a USBL, even if these updates are delayed; (ii) combining data obtained from other navigation sensors like a doppler velocity log (DVL), an attitude and heading reference unit (AHRS) or a pressure sensor; and (iii) allowing relative position updates between the AUV and the subsea panel.

### 3.1. Localization Filter

Vehicle and panel localization are achieved by merging, through an extended Kalman filter (EKF), the data gathered by a DVL, an AHRS, and a depth sensor as well as the USBL measurements from both the vehicle and the panel and relative updates between the vehicle and the panel computed by a vision-based system (see Section 3.3). The state vector proposed for this EKF is $x_{k}\;=\;{\left[x\;y\;z\;u\;v\;w\;l_{x}\;l_{y}\;l_{z}\;l_{\phi }\;l_{\theta }\;l_{\psi }\right]}^{T}$, where [xyz] is the vehicle position (in world coordinates), [uvw] is the vehicle linear velocity (in the vehicle body reference frame) and $\left[l_{x},l_{y},l_{z},l_{\phi },l_{\theta },l_{\psi }\right]$ is the panel position and orientation (in world coordinates).

A constant velocity kinematic model is used to determine how the vehicle will evolve from time k-1 to *k* while a constant position model is used by the landmark (i.e., the panel). The predicted state at time *k*, $x_{k}^{-}$ follows the equation:

(1) $$x_{k}^{-}=f\left(x_{k-1},n_{k-1},u_{k},t\right),$$

$$x_{k}^{-}=\left[\;\begin{array}{c} \left[\;\begin{array}{c} x_{k-1}\\ y_{k-1}\\ z_{k-1} \end{array}\;\right]+R\left(\phi_{k}\theta_{k}\psi_{k}\right)\left(\;\left[\;\begin{array}{c} u_{k-1}\\ v_{k-1}\\ w_{k-1} \end{array}\;\right]t+\left[\;\begin{array}{c} n_{u_{k-1}}\\ n_{v_{k-1}}\\ n_{w_{k-1}} \end{array}\;\right]\frac{t^{2}}{2}\right)\\ u_{k-1}+n_{u_{k-1}}t\\ v_{k-1}+n_{v_{k-1}}t\\ w_{k-1}+n_{w_{k-1}}t\\ l_{x_{k-1}}\\ l_{y_{k-1}}\\ l_{z_{k-1}}\\ l_{\phi_{k-1}}\\ l_{\theta_{k-1}}\\ l_{\psi_{k-1}} \end{array}\right].$$

where *t* is the time period, **u** = [ϕθψ] is the AHRS output, which determines the current vehicle orientation, **n** = [$n_{u}\;n_{v}\;n_{w}$] is a vector of zero-mean white Gaussian acceleration noise, and

(2) $$R\left(\phi_{k}\theta_{k}\psi_{k}\right)=\left[\begin{array}{ccc} cos\theta cos\psi & -cos\phi sin\psi +sin\phi sin\theta cos\psi & sin\phi sin\psi +cos\phi sin\theta cos\psi \\ cos\theta sin\psi & cos\phi cos\psi +sin\phi sin\theta sin\psi & -sin\phi cos\psi +cos\phi sin\theta sin\psi \\ -sin\theta & sin\phi cos\theta & cos\phi cos\theta \end{array}\right].$$

Four measurement updates are applied to the filter: DVL velocities ([u,v,w]), depth sensor ([z]), USBL updates ([x,y,z] or $\left[l_{x},l_{y},l_{z}\right]$), and landmark detections (i.e., relative position and orientation between the vehicle and the subsea panel). All of these updates are linear and follow the model:

(3) $$z_{k}=Hx_{k}+s_{k},$$

where $z_{k}$ is the measurement itself, H is the observation matrix that relates the state vector with the sensor measurement, and $s_{k}$ is the sensor noise. The filter is initialized when the AUV [x,y,z] and the panel $\left[l_{x},l_{y},l_{z}\right]$ positions are measured by the USBL system. It is worth noting that, to initialize the filter, the estimated panel orientation must be manually introduced by the user.

### 3.2. Delayed Updates

Because the localization filter is executed in real-time on-board the I-AUV, all sensor measurements are introduced as they arrive following Equation (3) except for USBL measurements. These updates contain information regarding the past: the vehicle or the panel are localized by the USBL system, placed at the surface, and this information is sent back to the vehicle through acoustics. Therefore, the vehicle receives this measure delayed between 2.0 to 10.0 s. USBL measures for the panel can be integrated directly in the filter because the panel is static, but vehicle measures must be projected into the present before doing the update. To do it, the vehicle has a database in which it keeps track of all vehicle position estimations for the last 10 s. When a vehicle USBL measure is received, the filter checks which was the estimated vehicle position at the time that this measure was done, each measure has a time stamp, and then computes the distance traveled from this moment to the present according to the positions in the database. The computed distance is added to the USBL measure and the update is done using the current time instead of the one in the USBL measure.

### 3.3. Panel Detection

Although incorporating USBL absolute position measurements, the accuracy in the vehicle/panel position will be on the order of some decimeters (i.e., around one meter according to USBL specifications and empiric tests in shallow water). Because the docking maneuver tolerates a maximum error of 10 cm, panel feedback is necessary. A vision-based algorithm that compares the images gathered by the vehicle’s front camera against an a priori known template of the panel is used to detect the panel and compute its relative position with respect to the vehicle. For each gathered image, a set of features is extracted and matched against the pre-computed set of features detected in the a priori known template. When a sufficient number of these features are matched, the position/orientation of the panel can be accurately estimated. The proposed algorithm uses the oriented features from accelerated segment test (FAST) and the binary robust independent elementary features (BRIEF) rotated (ORB) ([23]) feature extractors for its suitability for real-time applications. The ORB feature extractor detects key-points in the image. Due to the man-made nature of the docking panel, multiple key-points are detected in it. Compared with feature extractors such as scale invariant feature transform (SIFT) [24] and speeded-up robust features (SURF) [25], BRIEF allows real-time matching of key-points at higher image frame-rates.

Panel detection updates are applied using Equation (3), while the observation matrix H is defined as:

(4) $$H=\left[\begin{array}{cccc} -R{\left(\phi_{k}\theta_{k}\psi_{k}\right)}^{T} & 0_{3\times 3} & R{\left(\phi_{k}\theta_{k}\psi_{k}\right)}^{T} & 0_{3\times 3}\\ 0_{3\times 3} & 0_{3\times 3} & 0_{3\times 3} & I_{3\times 3} \end{array}\right],$$

where $I_{3\times 3}$ denotes the 3×3 identity matrix, $0_{3\times 3}$ denotes the 3×3 zero matrix, and R is defined in Equation (2).

## 4. Docking Maneuver

**Figure 2:**
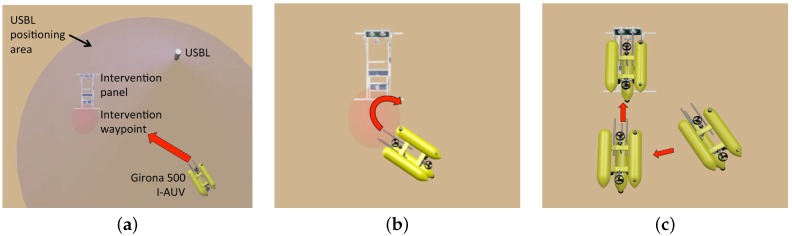
(**a**) Girona 500 I-AUV reaches a waypoint in front of the panel within the ultra-short base line (USBL) accuracy; (**b**) the autonomous underwater vehicle (AUV) centers the panel in its field of view (FoV) in order to obtain several panel detections to improve its relative position; and (**c**) two waypoints are computed to complete the docking maneuver. If the second waypoint is reached, the vehicle pushes forward to finalize and keep the docking.

To carry out an intervention mission using a fixed-base manipulation approach, the first task to perform is to approach the subsea panel and dock to it. The docking maneuver is composed of three steps: (i) approaching the panel; (ii) improving the relative position between the vehicle and the panel using the vision-based system; and (iii) docking. Figure 2 shows a graphical representation of these three steps.

Once the filter is initialized (see Section 3), the vehicle navigates towards a waypoint placed in front of the panel. Once there, the AUV centers the panel in its field of view (FoV) in order to obtain several panel detections to improve the relative position between the AUV and the panel. The third step consists of facing the panel and approaching it slowly until the probes are almost inside the funnel shaped receptacles. To do it, two waypoints are automatically computed, one at 1.5 m and another at 0.7 m from the panel center where the vehicle probes will be almost inside the funnel shaped receptacles (see Figure 2c). The vehicle is requested to move towards these waypoints using a holonomic 4 DoF controller (*x*, *y*, *z*, *ψ*). A forward thrust will be exerted to conclude the docking maneuver and to keep the vehicle attached to the panel. Once the manipulation is completed, the vehicle inverts the forward thrust to undock.

**Figure 3:**
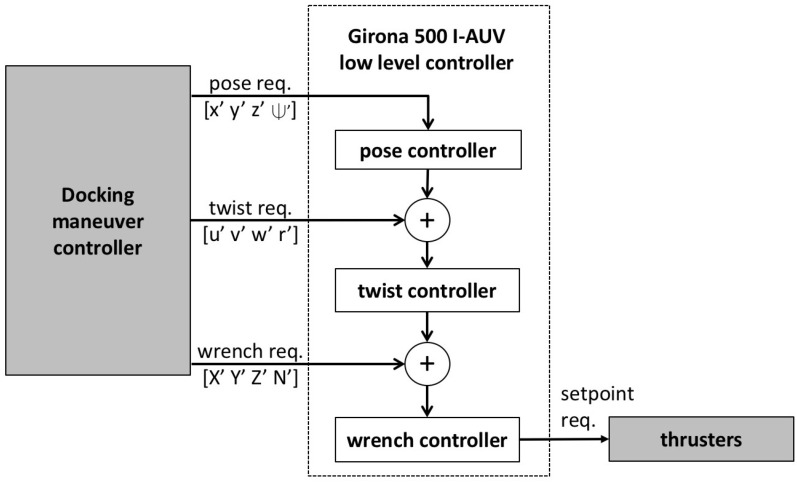
Girona 500 I-AUV low level cascade control scheme.

Girona 500 I-AUV has three different control modes available: pose (position + orientation), twist (linear + angular velocities), and wrench (force + torque). A three-stage cascade control scheme is used to combine them (see Figure 3). In the first stage, the pose controller receives desired poses and transforms them into twist requests. It consists of a 4 DoF proportional-integral-derivative (PID) controller.

(5) $$\nu^{\prime }\left(t\right)=K_{p}\left(e\left(t\right)+\frac{1}{T_{i}}\int_{0}^{t}e\left(t\right)dt+T_{d}\frac{d}{dt}e\left(t\right)\right),$$

where e(t) is the error for each DoF $\left[e_{x}\;e_{y}\;e_{z}\;e_{\psi }\right]$ computed as

(6) $$\begin{array}{ccc} \left[\begin{array}{c} e_{x}\\ e_{y}\\ 1 \end{array}\right] & = & \left(\begin{array}{cc} R2{\left(\psi \right)}^{T} & -R2{\left(\psi \right)}^{T}\;\left[\begin{array}{c} x\\ y \end{array}\right]\\ 0_{1\times 2} & 1 \end{array}\right)\left[\begin{array}{c} x^{\prime }\\ y^{\prime }\\ 1 \end{array}\right],\\ e_{z} & = & z^{\prime }-z,\\ e_{\psi } & = & normalized(\psi^{\prime }-\psi ), \end{array}$$

where [xyzψ] is the current vehicle position, $\left[x^{\prime }\;y^{\prime }\;z^{\prime }\;\psi^{\prime }\right]$ the desired one, R2 is a 2D rotation matrix and the normalized(x) function wraps the angle *x* between -π and *π*. The second stage, the twist controller, receives desired twists ($\nu =[u^{\prime }\;v^{\prime }\;w^{\prime }\;r^{\prime }]$) and transforms them into wrench requests. It is composed of a 4 DoF (PID) controller and a 4 DoF open loop model working in parallel:

(7) $$\tau^{\prime }=PID\left(\nu ,\nu^{\prime }\right)+Model\left(\nu^{\prime }\right).$$

The whole nonlinear model of the Girona 500 I-AUV and their parameters are defined and identified in [26]. For the reader’s convenience, they are also included here:

(8) $$\begin{array}{c} m_{u}\dot{u}-m_{v}vr+m_{w}wq+\left(W-B\right)sin\left(\theta \right)-X_{u}u-X_{|u|u}\left|u\right|u=X, \end{array}$$

(9) $$\begin{array}{c} m_{v}\dot{v}-m_{w}wp+m_{u}ur-\left(W-B\right)cos\left(\theta \right)sin\left(\phi \right)-Y_{v}v-Y_{|v|v}\left|v\right|v=Y, \end{array}$$

(10) $$\begin{array}{c} m_{w}\dot{w}-m_{u}uq+m_{v}vp-\left(W-B\right)cos\left(\theta \right)cos\left(\phi \right)-Z_{w}w-Z_{|w|w}\left|w\right|w=Z, \end{array}$$

(11) $$\begin{array}{c} I_{r}\dot{r}-N_{r}r-N_{|r|r}\left|r\right|r=N, \end{array}$$

where $m_{u}=249.5384$, $m_{v}=367.7126$, $m_{w}=659.9799$, $I_{r}=74.9024$, W-B=-37.3058, $X_{u}=-42.4181$, $X_{|u|u}=-125.3578$, $Y_{v}=-75.7673$, $Y_{|v|v}=-447.6195$, $Z_{w}=-44.0561$, $Z_{|w|w}=-325.0138$, $N_{r}=-20.5833$, and $N_{|r|r}=-60.9373$.

However, for the velocity controller, the model used for each degree of freedom is not this highly non-linear model but a polynomial function that has been adjusted fitting empirically gathered data. In the last stage, the wrench controller receives desired wrenches ($X^{\prime }\;Y^{\prime }\;Z^{\prime }\;N^{\prime }$) and transforms them into thruster set points. It contains a thruster allocation matrix that distributes the wrench vector to be achieved by the vehicle among the available thrusters according to its location in the vehicle. The wrench controller also contains a thruster model that transforms the force per thruster into revolutions per minute (RPMs) or voltage depending on the thruster driver.

The docking maneuver equally interacts with all the controllers: it uses a combination of pose ($z^{\prime }\;\psi^{\prime }$) and twist ($u^{\prime }$) requests to approach the panel, only pose requests ($x^{\prime }\;y^{\prime }\;z^{\prime }\;\psi^{\prime }$) for the docking, and pose ($z^{\prime }\;\psi^{\prime }$) and wrench ($X^{\prime }$) requests to complete and keep the docking as well as to undock.

## 5. Valve and Connector Detection

**Figure 4:**
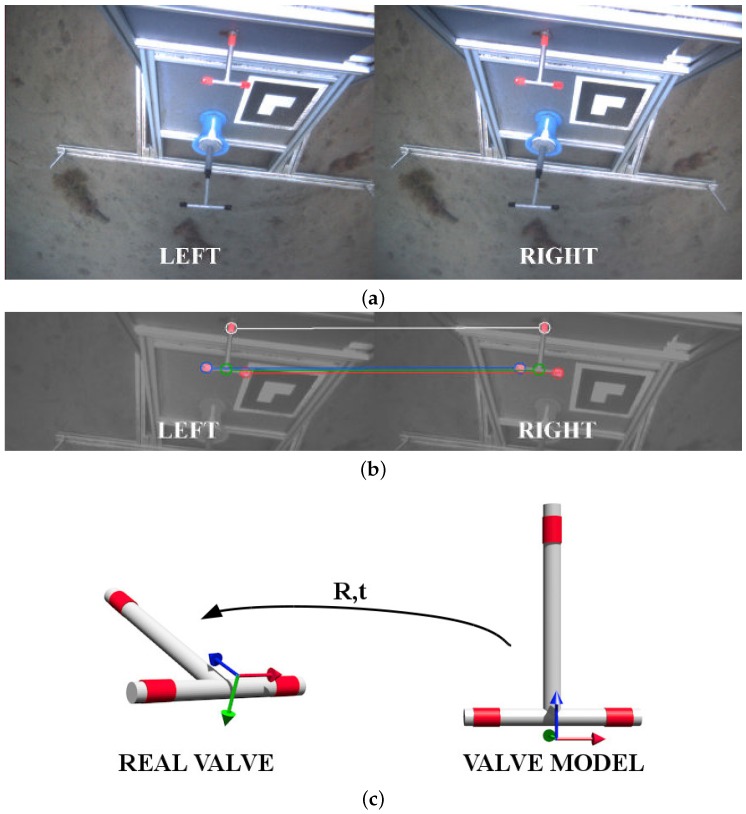
Valve detection procedure: (**a**) original stereo pair; (**b**) blob detection and matching; and (**c**) rigid transformation estimation.

The detection of the valve and the connector is accomplished by using a stereo camera placed in the Girona 500 I-AUV bottom hull. This camera is pointing downwards and slightly backwards in order to focus both valve and connector position once the vehicle is docked. Figure 4a shows how these two objects are seen by the vehicle during sea trials.

Both detections are targeted to deep ocean stations where divers cannot reach and waters are usually see-through and clear. Once the vehicle is docked, the targets are in a one-meter range from the camera. Therefore, the impact of turbidity on the image detection algorithms is minimal.

### 5.1. Valve Detection

In order to detect the valve position and rotation, red markers are used on the three valve ends. A color-based detection in the hue, saturation, and value (HSV) space [14], using both hue and saturation histograms, is carried out once a histogram training procedure is completed. For the two stereo frames (left and right), the three red blobs are extracted and its centroids are then computed (see Figure 4b). These centroids are matched between left and right and triangulated using the stereo camera model, resulting in three 3D points. An additional point is added on the cross of the T-shape valve by computing the midpoint of the segment between left and right points of the valve. Therefore, at the end of this process, four 3D points are obtained.

To estimate the optimal rigid transformation from the camera to the valve, a model of the valve, defined by the same four points, is placed at the origin of the camera frame (see Figure 4c). Then, $p^{\prime }=R\cdot p+t$ is applied, p being the points of the valve model and $p^{\prime }$ the points detected by the color-based algorithm. *R* is a 3×3 rotation matrix and *t* a 3D translation vector. Because the a priori known 3D model is defined at the camera frame origin, the transformation defined by (R|t) is the homogeneous transformation of the detected valve with respect to the camera. The equation is solved using singular value decomposition (SVD) as explained below.

*c* and $c^{\prime }$ being the centroids of p and $p^{\prime }$, the points can be translated to the same origin for SVD computation:

(12) $$H=\sum_{i=1}^{N}\left(p_{i}^{\prime }-c^{\prime }\right)\left(p_{i}-c\right),$$

(13) [U,S,V]=SVD(H),

(14) $$R=V\cdot U^{⊤},$$

where *N* is the number of points, and $p_{i}$ and $p_{i}^{\prime }$ represent the *i*-th point of the corresponding dataset. The translation can be computed applying $t=-R\cdot c+c^{\prime }$.

Finally, since the valve model is located at the camera frame, the camera to valve transformation (${}^{c}T_{v}$) is composed as ${}^{c}T_{v}=\left(R|t\right)$.

### 5.2. Connector Detection

**Figure 5:**
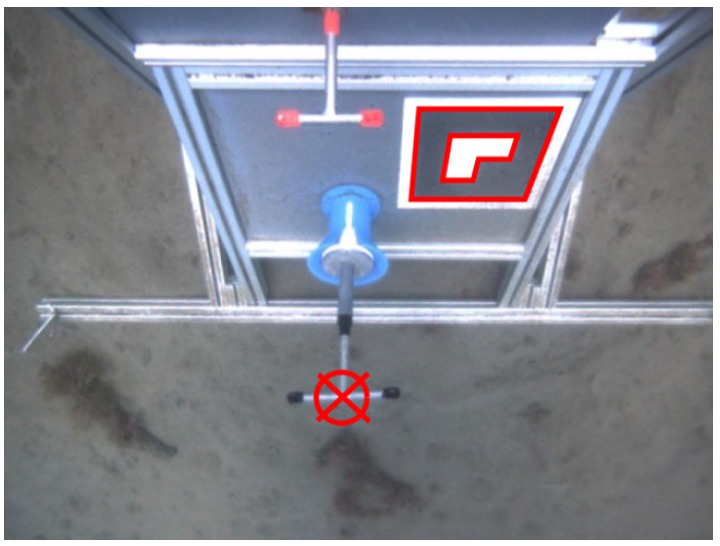
The ART marker is detected for an estimation of the position of the hot stab connector.

The connector detection method uses a marker from the augmented reality toolkit (ART) [27] placed next to the hot stab (see Figure 5). ART provides a library with marker detection methods for monocular cameras able to estimate the position and orientation of a marker giving only its dimensions. The transformation between the marker and the connector, named ${}^{m}T_{h}$, is static and calibrated off-line. Therefore, the transformation between the camera and the hot stab connector is obtained by ${}^{c}T_{h}\;={\;}^{c}T_{m}\cdot^{m}T_{h}$, where ${}^{c}T_{m}$ is the transformation between the camera and the marker.

Notice that the relative position of the connector to the valve is also fixed. Thus, if the marker is occluded by the manipulator, the position of the connector can be estimated through the position of the valve. The camera to connector transformation is computed by ${}^{c}T_{h}\;={\;}^{c}T_{v}\cdot^{v}T_{h}$, where ${}^{v}T_{h}$ is static and calibrated off-line. However, this method is less accurate than using the marker, especially when the valve is oriented vertically. In the opposite case, when the valve is occluded by the manipulator, the camera–valve transformation cannot be estimated using the marker since it is impossible to know the valve orientation, and, therefore, the robot cannot proceed with the manipulation.

## 6. Arm Control

The manipulator used for the intervention is the light-weight ARM5E [28] (ECA group). It is a 4 DoF arm with a T-groove gripper that makes the manipulation of the T-shape handles easier. This arm has been attached to the bottom of the Girona 500 AUV.

The arm uses hall-effect sensors, located in the electrical motors, to know the relative position of each joint. Thus, it is needed to initialize the joints prior to each intervention. To this aim, each joint is moved individually until reaching its physical limit. During the movements, the current consumed by the joint is read. Once the current reaches a threshold, it means that the joint has reached its limit. Then, the joint is stopped and this relative position is set to the zero position. Then, the hall effect sensors are used to track the joint angles.

Nonetheless, it is quite common in robotic arms that kinematic errors appear due to bad initialization or miscalibration of the joints. In order to correct these problems, a visual servoing approach has been developed. This solution is able to calculate, in an autonomous way, the real values of the arm joints every time that an ART marker, placed on the top of the gripper, is detected by a camera.

**Figure 6:**
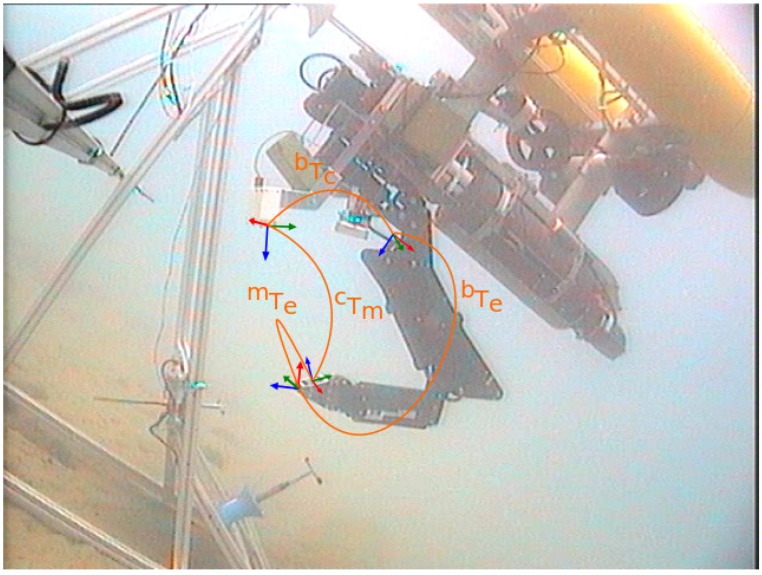
Girona 500 I-AUV at sea performing a fixed-base manipulation with all the manipulation system frames and transformations marked.

The methodology needs to go through an initialization phase. In this phase, the transformation between the camera and the base of the arm (${}^{b}T_{c}$) is calculated (see Figure 6). For that, the arm is moved to a predefined position where the camera can clearly see the marker. Then, the aforementioned library provided by the ART is used to determine the pose of the marker with respect to the camera (${}^{c}T_{m}$). At that time, the relationship between the base of the arm and the end-effector (${}^{b}T_{e}$) is calculated using the arm forward kinematics. The third transformation used to initialize the visual servoing approach is the static and a priori known transformation between the marker and the end-effector (${}^{m}T_{e}$). As a result, the ${}^{b}T_{c}$ transformation can be obtained as follows: ${}^{b}T_{c}\;={\;}^{b}T_{e}\cdot {{(}^{c}T_{m}\cdot^{m}T_{e})}^{-1}$.

Once the algorithm is initialized, the manipulation can start. Henceforth, each time the marker is detected, the estimated transformation between the base of the arm and the end-effector is computed using: ${}^{b}T_{e}\;={\;}^{b}T_{c}\cdot^{c}T_{m}\cdot^{m}T_{e}$.

Then, the estimated values of each joint (*q*) can be calculated using the inverse kinematics of the arm:

(15) $$q=IK{(}^{b}T_{e}).$$

Next, the difference between the estimated joint values and the internal ones are computed:

(16) $$offset_{\left[1,\cdots ,n\right]}=estimated_{\left[1,\cdots ,n\right]}-internal_{\left[1,\cdots ,n\right]}.$$

Then, each time the system needs to know the joint positions, the current offset is added to the internal joint values. This approach reduces the arm inaccuracies, ensuring the pose consistency between the end-effector and the arm base. If, during a period of time, the camera cannot detect the marker, the offset remains constant. It means that some errors due to miscalibration can arise, but at the moment the camera detects the marker again, these errors are cancelled.

## 7. Valve and Connector Manipulation

When the vehicle is docked, the object detections are available, and the visual servoing algorithm is initialized, the manipulation begins. This manipulation consists of turning a valve and plug in/unplug a hot stab connector. Once the object that is going to be manipulated is detected, the system knows its pose relative to the camera (${}^{c}T_{o}$). Thus, in order to know that pose with respect to the base of the arm (${}^{b}T_{o}$), it has to calculate ${}^{b}T_{o}\;={\;}^{b}T_{c}\cdot^{c}T_{o}$.

Next, a basic grasp planning methodology has been used. It consists of generating an end-effector path by placing waypoints with respect to the object: the pre-manipulation, manipulation and post-manipulation waypoints for the valve, the object transition waypoint and the pre-manipulation, manipulation, and unplugging and plugging waypoints for the connector. Following this path, the arm avoids collisions with the panel, whose shape is already known, while it reaches the object in a proper orientation.

In order to reach the next waypoint, the system calculates the Cartesian distance ($x_{e}$) between the end-effector and this waypoint. Then, this distance is multiplied by the pseudo-inverse of the arm Jacobian at the end-effector ($J_{e}^{+}$) to obtain the joint velocities ($\dot{q}$) that will drive the end-effector to the waypoint:

(17) $$\dot{q}=J_{e}^{+}\cdot x_{e}.$$

Due to the limitations of the arm used, it has just 4 DoF, and the orientation in which the waypoints are reached is not taken into account. Thus, the last three rows of the Jacobian, which define the orientation, are set to zero.

## 8. Results

The goal of this article was to demonstrate how an I-AUV is able to autonomously dock into an adapted underwater panel and, once docked, perform a fixed-base manipulation that consists of turning a 1/4 valve and plugging in or unplugging a hot stab connector. To the best of the authors knowledge, this kind of autonomous intervention has never been demonstrated with a light-weight I-AUV.

**Figure 7:**
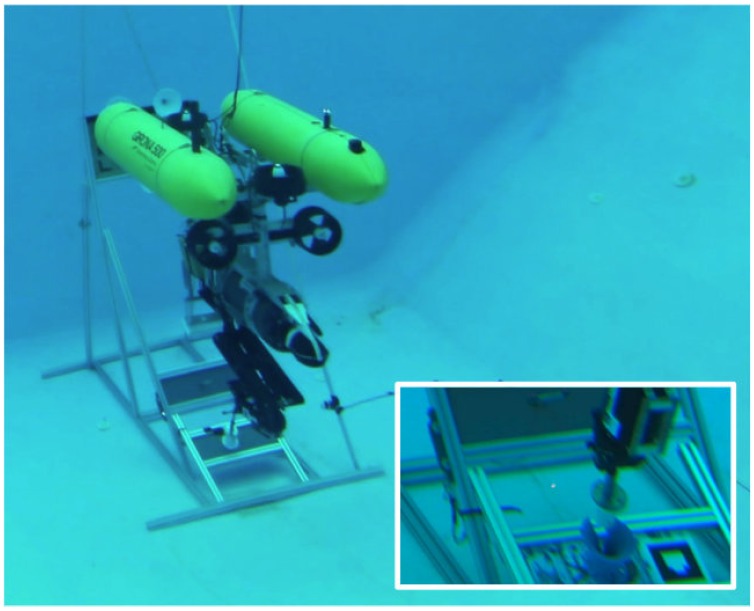
Girona 500 I-AUV docked in a subsea panel unplugging a hot stab connector in a water tank.

To validate the algorithms involved in this task as well as the overall autonomous intervention mission, two scenarios have been defined. In the first one, the mock up panel designed in Section 2 has been deployed in a water tank of 16 × 8 × 5 m and a Seaeye MCT1 thruster (SAAB Seaeye LTD.) has been placed next to it in order to artificially generate water currents. In the second scenario, the mock up panel has been deployed at a harbour area in St. Feliu de Guixols (Spain). In both scenarios, the Girona 500 I-AUV [21] equipped with a passive docking system, consisting of three probes and an ECA ARM5E manipulator with 4 DoF [28] has been used (see Figure 6 and Figure 7). Two cameras have also been mounted on the vehicle: one looking forward to estimate the panel pose (see Section 3), and the other pointing down to detect the intervention objects to be manipulated and to improve the manipulator’s end-effector pose estimation (see Section 5 and Section 6).

To test the reliability of the docking maneuver, a series of systematic tests have been conducted in the water tank using the external Seaeye MCT1 thruster to generate controlled water currents. In the water tank environment, the USBL system was not mounted, and consequently each trial was started from a position in which the intervention panel was within the vehicle’s camera FoV. To initialize the navigation filter, the vehicle position was set to x=0.0 and y=0.0 while panel detection measurements were used to initialize the panel position and orientation relative to the AUV. The docking maneuver was performed as described in Section 4 avoiding the approaching step. This test was repeated 12 times with different levels of water current: six tests were done without any perturbation and six more setting the perturbation thruster between 30% and 70% of its maximum 14 kg thrust. The I-AUV was able to successfully dock 11 times (>90%) and the precision achieved by the vehicle, according to its localization system, when the vehicle probes should be aligned and nearly touching the funnels in the panel, was: $\sigma_{x}=2.07$ cm, $\sigma_{y}=3.76$ cm, $\sigma_{z}=1.9$ cm, and $\sigma_{\psi }=0.76^{\circ }$. These errors were small enough to achieve the mechanical coupling between the vehicle probes and the funnels in the panel when the I-AUV pushed forward. It takes 115 s, on average, to complete the docking maneuver. After analyzing the results, the only registered failure seems to be caused by a CPU overload that caused a performance drop in the low level controller. To solve this issue, the localization filter was translated from Python to C++ and the old Core2Duo CPU (Intel Corporation, Santa Clara, CA, USA) used in the Girona 500 I-AUV was replaced by a more powerful i7 (Intel Corporation, Santa Clara, CA, USA).

The docking was also tested at sea adding the USBL system to localize both the AUV and the panel. The external thruster used to generate water currents was removed for logistic reasons. Six trials were attempted, including the approaching phase, and five of them were successful. Despite the AUV navigation system being able to drive the AUV at 1.5 m in front of the subsea panel ($0.78\sigma^{2}$), due to sunlight reflections and water turbidity issues, the vision-based system was unable to detect the panel on one occasion, aborting the whole intervention.

**Figure 8:**
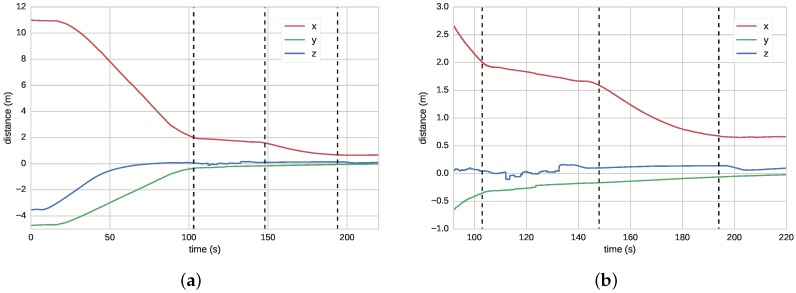
(**a**) vehicle position, estimated in real-time by the on-board localization filter, with respect to panel axes during a docking maneuver; and (**b**) zoom of the last part of the experiment shown in (**a**).

Figure 8 shows the I-AUV position with respect to the panel center while performing the docking maneuver (see Figure 1a to see the panel axes). Figure 8a shows the four steps involved in this maneuver: approaching the panel, centering the vehicle in front of the panel while acquiring vision-based updates, putting the vehicle probes inside the panel funnels, and pushing forward to conclude the docking. Figure 8b shows a zoom of the last part of this maneuver. It can be seen how the first vision-based updates appear at the end of the approaching phase. In the second phase, panel updates caused small corrections in the vehicle estimated position, especially on the *y*-axis where small jumps can be seen. It is worth noting how between seconds 200 to 210 there is also a small perturbation on all the axes due to the mechanical coupling between the AUV and the panel.

**Figure 9:**
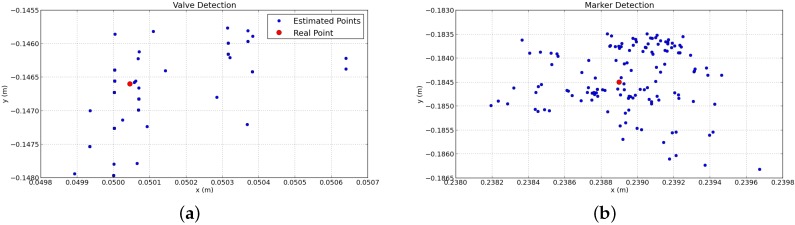
Valve detection (**a**) and AR Marker (**b**) estimations on the *x*/*y* plane.

Once the I-AUV was docked, the detection of panel elements started. Both valve and connector detections were influenced by light changes and occlusions. Regarding the valve detection, the size and shape of the marks changed depending on the viewpoint, and the detected centroid was shifted from its actual center, causing small errors. The AR Marker detection showed more reliability when the marker was closer to the camera, so that the total size in pixels was bigger and therefore the computation of its pose was more precise. To estimate the repeatability of these errors, a static test was performed keeping the I-AUV docked and without moving the I-AUV and the manipulator for 30 s. Figure 9 illustrates the valve/connector pose estimations during this static test. The fewer number of points for the valve detection plot is due to the low frequency rate of this algorithm. The repeatability error for the valve detection was 0.57 mm with a standard deviation of 0.53 mm, whilst for the AR Marker, the average error was 0.9 mm with a standard deviation of 0.79 mm.

To test the robustness of the manipulation task, several tests turning the valve and plugging in/unplugging the connector were performed. In order to make the demonstrations more challenging and test if the system was able to dynamically adapt to changes, before the plugging phase, and with the connector already grasped, the vehicle performed an undocking and a docking maneuver. This additional step made the arm position change slightly with respect to the panel elements due to the mechanical tolerances of the docking system. Therefore, the vision system was required to calculate the position of the valve and the connector continuously.

Like the docking maneuver, the manipulation was tested first in a water tank and then in the sea, obtaining an ∼80% and ∼60% of success, respectively. False detections in the vision-based detection algorithms, mostly caused by illumination problems, produced this decline in the success rate at sea. Preliminary tests including docking and intervention in a water tank can be found at [29].

**Figure 10:**
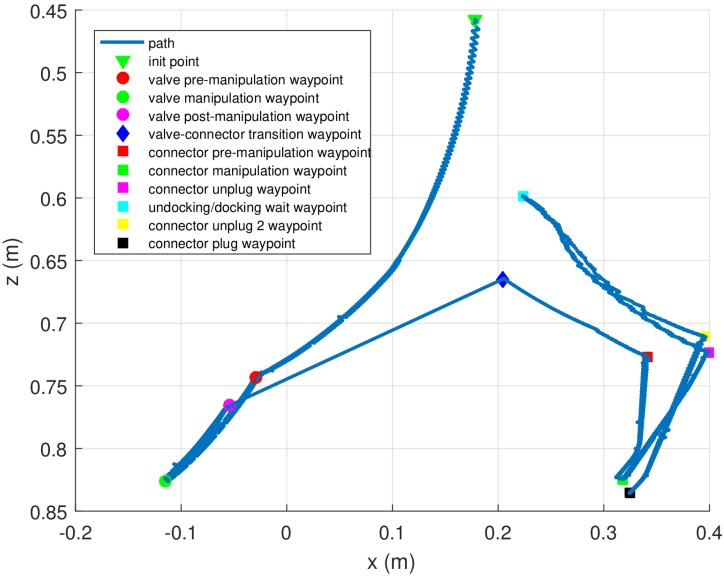
End-effector Cartesian trajectory with respect to the base of the arm during the intervention.

Figure 10 shows the Cartesian trajectory followed by the end-effector in a complete intervention in which the I-AUV docks, manipulates the valve, unplugs the hot stab, undocks, docks again from the panel vicinity and plugs in the hot stab connector. The end-effector tries to reach several waypoints defined with respect to the estimated pose of the object of interest. In the figure, it can be seen that the pose where the connector is with respect to the base of the arm is not exactly the same after the two docking maneuvers (i.e., connector manipulation and connector plug waypoints).

**Figure 11:**
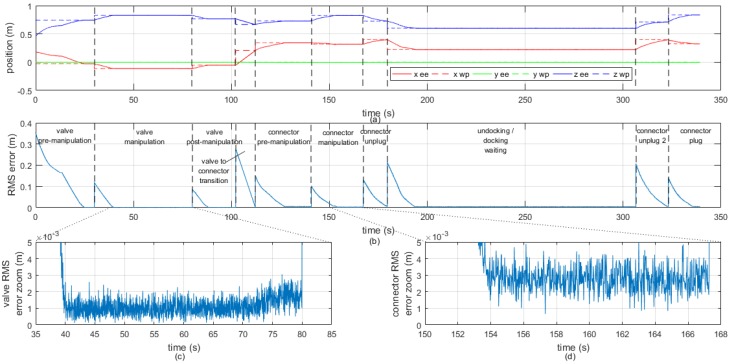
End-effector Cartesian trajectory decomposed in its three components: (**a**) Root means square (RMS) error of the end-effector position with respect to the next waypoint (**b**) and zoom of the RMS error (**c**,**d**).

Figure 11a illustrates the time evolution of each component of the 3D end-effector trajectory represented in Figure 10, together with the waypoints which define the trajectory to follow. The root mean square (RMS) error of the end-effector position with respect to the next waypoint to reach is shown in Figure 11b. Figure 11c,d show a zoom of the RMS error in order to evince the noise that appears due to the repeatability error of the vision system. However, this high frecuency error does not affect the end-effector trajectory because the dynamics of the manipulator is much slower. It is worth noting that the waypoints were reached with an average precision of 2 mm between the estimated position of the end-effector and the desired waypoint.

Errors in the manipulation phase were produced by inaccuracies in: the vision-based object detection system, the end-effector visual position estimation, the arm controller, and the arm calibration process. Although all these inaccuracies are individually lower than 3 mm, their combination produces up to 2 cm errors. In order to cope with these larger errors, the hot stab socket was designed with a funnel shape and with a flexible handle, by using a spring, and the end-effector was V-shaped to mechanically simplify both the turning and plugging in/unplugging tasks.

Two videos showing the water tank and the sea trials in which the I-AUV docks and performs an autonomous intervention can be seen at VIDEO1 and VIDEO2 included in the Supplementary Materials section.

## 9. Conclusions

In this article, several systems have been integrated in the context of a subsea panel docking and intervention mission. Several tests have been done with the light-weight I-AUV Girona 500 first in a water tank and later at sea. Designing and building an AUV-friendly intervention panel has been a key element to simplify the docking maneuver as well as a fixed-based manipulation from an autonomous intervention vehicle.

The updates computed by a feature-based vision algorithm to estimate the panel pose have been combined with the navigation data gathered by the I-AUV sensors and the delayed USBL updates in a localization filter. This solution has demonstrated its reliability to estimate both the vehicle and the panel position. Other vision-based algorithms have also been developed to estimate the position of the elements of interest in the panel, the valve and the connector, as well as to improve the pose estimation of the end-effector through visual servoing techniques. It must be said that AR Marker-based solutions have been more robust than color-based approaches despite the repeatability error being slightly bigger. While the former have worked out of the box both in the water tank and at sea, the latter have required adjustments due to light changes, especially at sea. Initial problems with the position of the manipulator’s end-effector have been partially solved with the inclusion of a visual servoing algorithm that has substantially improved the arm accuracy. Due to all these vision-based algorithms running in parallel, computation power is quite demanding, but it can be handled by any current state-of-the-art CPU.

Most of the problems faced during trials have been related with vision-based systems (i.e., panel, valve, connector, and end-effector detection algorithms) or with limitations in the manipulator. Vision-based problems have been especially relevant at sea where the visibility and illumination, due to water turbidity and sun reflections, were worse than in the water tank. To overcome these issues, we plan to replace the passive markers by active light beacons with specific blinking frequencies. Preliminary results have been proven satisfactory, being able to locate the light beacons at sea up to several meters [30]. Problems related with the manipulator can be summarized in two points: the arm uses hall-effect sensors that provide only relative positions for each joint, and its working space is very limited. Underwater manipulators for light weight AUVs are in their early stages, and there is still much room to improve. However, with the implemented end-effector visual servoing algorithm and few adaptations in the subsea panel, we have relieved these two problems.

To conclude, satisfactory results have been obtained both in a water tank and at sea with an average visibility of 2–3 m and performing only basic adaptations to the subsea panel. In the future, acoustic and active light beacons will be further developed to provide a higher degree of reliability in low visibility conditions.

## Supplementary Materials

VIDEO1 I-AUV Docking and Intervention in a Subsea Panel in a water tank (accessible online at: https://www.youtube.com/watch?v=SL-WMBdjxRg); VIDEO2 I-AUV Docking and Intervention in a Subsea Panel at sea (accessible online at: https://www.youtube.com/watch?v=xA2SGLi5TYg&feature=youtu.be).

## Abbreviations

The following abbreviations are used in this manuscript:

AHRS: attitude and heading reference unit
AUV: autonomous underwater vehicle
BRIEF: binary robust independent elementary features
DoF: degree of freedom
DP: dynamic positioning
DVL: doppler velocity log
EKF: extended Kalman filter
FAST: features from accelerated segment test
FoV: field of view
HROV: hybrid ROV
HSV: hue, saturation, and value
IMR: inspection maintenance and repair
I-AUV: intervention AUV
ORB: oriented FAST and rotated BRIEF
PID: proportional-integral-derivative
RMS: root mean square
ROV: remotely operated vehicle
RPM: revolutions per minute
SAUVIM: Semi-Autonomous Underwater Vehicle for Intervention Missions
SIFT: scale invariant feature transform
SURF: speeded-up robust features
SVD: singular value decomposition
USBL: ultra-short base line
